# Ideals of Education

**Published:** 1906-01-13

**Authors:** 


### The Hospital.

A Journal of

The Medical Sciences and Hospital Administration.

Vol. XXXIX.—No. 1,007. SATURDAY, JANUARY 13, 1906.

### Ideals of Education.

In all ages, national ideals of education have varied with time and circumstance. It is said that, among the ancient Persians, lads were taught "to ride, to draw the bow, and to speak the truth." In a comparatively modern Italy, but before the regeneration wrought by the genius of Cavour, we were once told by a schoolboy that his education comprised "dancing, singing, and pretty behaviour." Among ourselves, in the near future, if we may place reliance upon a report of an address delivered by Lord Roberts to the Association of Assistant Masters in Secondary Schools, and a report of the proceedings at a Conference of teachers from the elementary and secondary schools of the London County Council, the two main subjects will be military drill and rifle shooting on the one hand, and instruction in the English language on the other. We shall welcome them both; and the second scarcely more for its own sake than on account of the extent to which it will be conducive to the attainment of the third and most difficult, as well as the most important, of the Persian accomplishments. To speak the truth is never easy, but it becomes impossible when attempted in a language which is not understanded of the speaker. When an area-sneak runs away with some spoons, and the paragraphist of an evening paper describes the theft as "mysterious," the latter personage does not necessarily intend to be mendacious. He does not know the meaning of the word, and all he intends to convey by it is that he is not acquainted with the name and address of the culprit. But it unfortunately happens that such ignorance supplies the intellectual pabulum of a very large majority of the English people; and hence that the words and phrases of our noble language are misapplied in common speech to the very confines of absolute unintelligibility. Even Lord Roberts himself is open to some criticism in this respect. He told his hearers in one sentence that the managers of 12 schools expressed themselves as being "definitely opposed" to the movement which he advocated; and said in the next that "there could be no doubt" of the great advantages which would accrue from its adoption. Obviously there is and can be much doubt, although Lord Roberts himself does not share it, and although we, for our parts, are much disposed to agree with his opinions, we think that a large proportion of the energies which are now expended upon games of a useless character might be profitably diverted to a pursuit which would qualify those following it to take some part in the defence of their country. Lord Roberts proposes to make room for drill and rifle shooting by the abandonment, if necessary, of classical subjects, which he describes as of doubtful help in after-life to the individual and of none whatever to the State. He would substitute for them the development of the boy's body and intelligence by military training and the study of modern history, science, and geography, and the strengthening of his moral character by habits of discipline and obedience. So far as these habits of discipline and obedience.

From the point of view with which we, as medical journalists, are chiefly concerned, we may perhaps adopt Lord Roberts's very positive phraseology, and may declare there to be "no doubt" that the general adoption of his recommendations would be likely to exert a beneficial effect upon the sanitary condition of the population. Military drill, even if it has no other claim, at least provides opportunities, such as are denied to the average civilian, of attaining a high standard of physical development. And any improvement of national physique resulting from the universal training which Lord Roberts suggests, would almost certainly diminish the national liability to pulmonary phthisis. Taken alone, it would not be sufficient to overcome powerfully predisposing causes, as was abundantly shown in the mortality from the disease which prevailed in the regiments of guards, before their quarters were improved by the light of modern science; but it would unquestionably exert a very considerable influence in the right direction. In this, as in many other cases, it is probable that the great bulk of the juvenile population would be more easily influenced indirectly than directly. It would be theoretically possible to have classes for respiratory and other movements as a part of ordinary education; but it is practically certain that little or no good would be likely to accrue from them. Whatever may be the case with philosophers of the Spencerian school, "militarism" always appeals to boys of healthy nature, in whom the fighting instincts of the race have not been swamped by the desires and feelings which the apostle describes generically as "covetousness." To such, the idea of being trained to take their part in national defence would invariably appeal, and would impart a living interest to the instruction and a desire to excel in what was being taught. On the same ground, it follows that drill should never be employed as a punishment; or, in other words, that participation in it should be regarded as a privilege, and premature dismissal as a disgrace. On such conditions we should certainly be disposed to agree with Lord Roberts as to its probable value to the population, and to join with him in urging upon parents and upon teachers the good results which might be expected to follow from its adoption.

